# Neuroplastic Mechanisms of Acupuncture in Post‐Stroke Motor Recovery: A Randomized Multimodal MRI Trial

**DOI:** 10.1002/cns.70955

**Published:** 2026-06-03

**Authors:** Xin Yu, Nana Zhao, Yuchen Liu, Shizhong Zheng, Dongtao Luo, Qianji Chen, Fufu Zeng, Jiao Chen, Yanfei Jia, Yihuai Zou

**Affiliations:** ^1^ Department of Acupuncture Shenzhen Luohu Hospital of Traditional Chinese Medicine (Shanghai University of Traditional Chinese Medicine, Shenzhen Hospital) Shenzhen China; ^2^ Department of Neurology Beijing University of Traditional Chinese Medicine, Dongzhimen Hospital Beijing China

**Keywords:** acupuncture, neuroimaging, neuroplasticity, stroke

## Abstract

**Background:**

Hemiplegia is a common motor impairment following ischemic stroke. Post‐stroke recovery involves extensive reorganization of structural and functional brain networks, which correlates with clinical deficits and recovery. While acupuncture has demonstrated safety and efficacy in improving motor function, robust neuroimaging evidence elucidating its central mechanisms remains limited. This study aimed to investigate the specific neuroplastic effects of acupuncture by focusing on its modulation of cortical gray matter volume (GMV) and multilayer dynamic functional network topology.

**Methods:**

Patients with stroke were randomly allocated in a 2:1 ratio to receive either true‐acupoint (TA) or sham‐acupoint (SA) over a two‐week intervention period. Clinical outcomes were assessed using the Fugl‐Meyer Assessment (FMA), Brunnstrom Scale, and the National Institutes of Health Stroke Scale (NIHSS). Neuroplastic changes were evaluated through GMV analysis and dynamic functional network metrics. Correlation analyses were performed to examine the relationships between post‐acupuncture improvements in clinical scores and changes in neuroimaging indices.

**Results:**

The results demonstrated that: (1) Clinically, post‐treatment improvements in NIHSS and FMA scores, but only the TA group exhibited significant gains in Brunnstrom scores, indicating superior motor recovery. (2) Functionally, TA modulated default mode network (DMN) dynamics, manifesting as significantly reduced disjointedness and a trend toward decreased flexibility. These changes correlated with better motor outcomes. No such modulatory effect was observed in the SA group. (3) Structurally, TA increased GMV in the right middle frontal gyrus, right postcentral gyrus, right angular gyrus, left superior parietal gyrus, left cerebellar Crus 1–2, 4–5, 7, bilateral middle occipital gyrus, superior temporal gyrus, angular gyrus of inferior parietal margin, dorsolateral superior frontal gyrus, inferior frontal gyrus of operculum, and cerebellar area 10. Increases in GMV in the right opercular inferior frontal gyrus, postcentral gyrus, and cerebellar region 10 were positively correlated with motor recovery in the TA group. No significant changes in GMV were detected in the SA group.

**Conclusions:**

TA treatment significantly alleviated neurological impairment and enhanced motor recovery. Its therapeutic effect is underpinned by specific neuroplastic changes: Functional modulation of DMN dynamics and structural promotion of gray matter plasticity in regions critical for sensorimotor and cognitive‐motor integration, which are essential for movement control.

## Graphical abstract

This randomized controlled trial found the true‐acupoint (TA) intervention to be superior to the sham‐acupoint (SA) intervention in improving motor recovery and neurological deficits post‐stroke. TA uniquely induces neuroplastic changes, evidenced by the modulation of default mode network dynamics and the promotion of gray matter plasticity in key regions responsible for sensorimotor and cognitive‐motor integration. These structural and functional alterations are correlated with superior clinical outcomes, offering new insights into acupuncture's central mechanism.

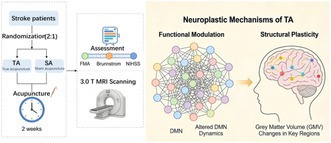

## 1 Introduction

Globally, stroke is the leading cause of Disability‐Adjusted Life Years (DALYs) in adults [1], of which ischemic stroke accounts for approximately 43% [2]. Post‐stroke hemiplegia not only seriously affects the quality of life of patients but also brings heavy economic pressure to the healthcare system and families. Acupuncture is a safe and effective complementary treatment for stroke. Its simplicity, low cost, and favorable side‐effect profile have facilitated widespread clinical adoption and are associated with positive patient outcomes [3]. The latest systematic review confirms the efficacy of acupuncture in improving neurological function [4]. However, further rigorously controlled, high‐quality randomized controlled studies are warranted to establish more robust evidence.

Over the past decades, the advent of functional Magnetic Resonance Imaging (fMRI) has significantly advanced our understanding of the brain. Previous studies have demonstrated abnormal functional connectivity within the sensorimotor network (SMN), the default mode network (DMN), and the cognitive control network in stroke patients [5, 6]. Conventional fMRI analysis typically treats the Blood Oxygen Level‐Dependent (BOLD) signal as static, which fails to capture the dynamic properties of functional networks. By contrast, dynamic analysis incorporates a temporal dimension and offers greater sensitivity in detecting functional alterations related to physiological or pathological states [7, 8]. Nevertheless, it remains unclear how acupuncture modulates the dynamic reconfiguration of brain networks following stroke.

Although brain structure is relatively stable compared to functional dynamics, recent structural Magnetic Resonance Imaging (sMRI) studies in stroke patients have revealed widespread cortical thinning with a distinctive topographic distribution [9]. However, the regulatory effects of acupuncture on the structure in patients with post‐stroke hemiplegia remain largely unexplored.

The neural mechanisms of motor function recovery are complex, involving functional and structural reorganization within the brain [10]. These neuroplastic changes may correlate with clinical neurological deficits and functional recovery outcomes [11, 12, 13]. sMRI visualizes brain structural remodeling with high resolution, and fMRI monitors functional recovery by recording BOLD signals at millisecond resolution. Integrating sMRI and fMRI data offers a comprehensive approach to elucidate the mechanisms by which acupuncture modulates post‐stroke motor dysfunction from complementary anatomical and functional perspectives.

In the current study, we combined multimodal MRI to investigate the neural mechanisms of acupuncture in stroke patients with unilateral motor impairment. We aimed to characterize the specific modulatory effects of acupuncture on both dynamic functional networks and static brain structure, thereby strengthening the evidence for its efficacy in treating post‐stroke motor dysfunction.

## 2 Methods and Analyses

### 2.1 Trial Design

**FIGURE 1:**
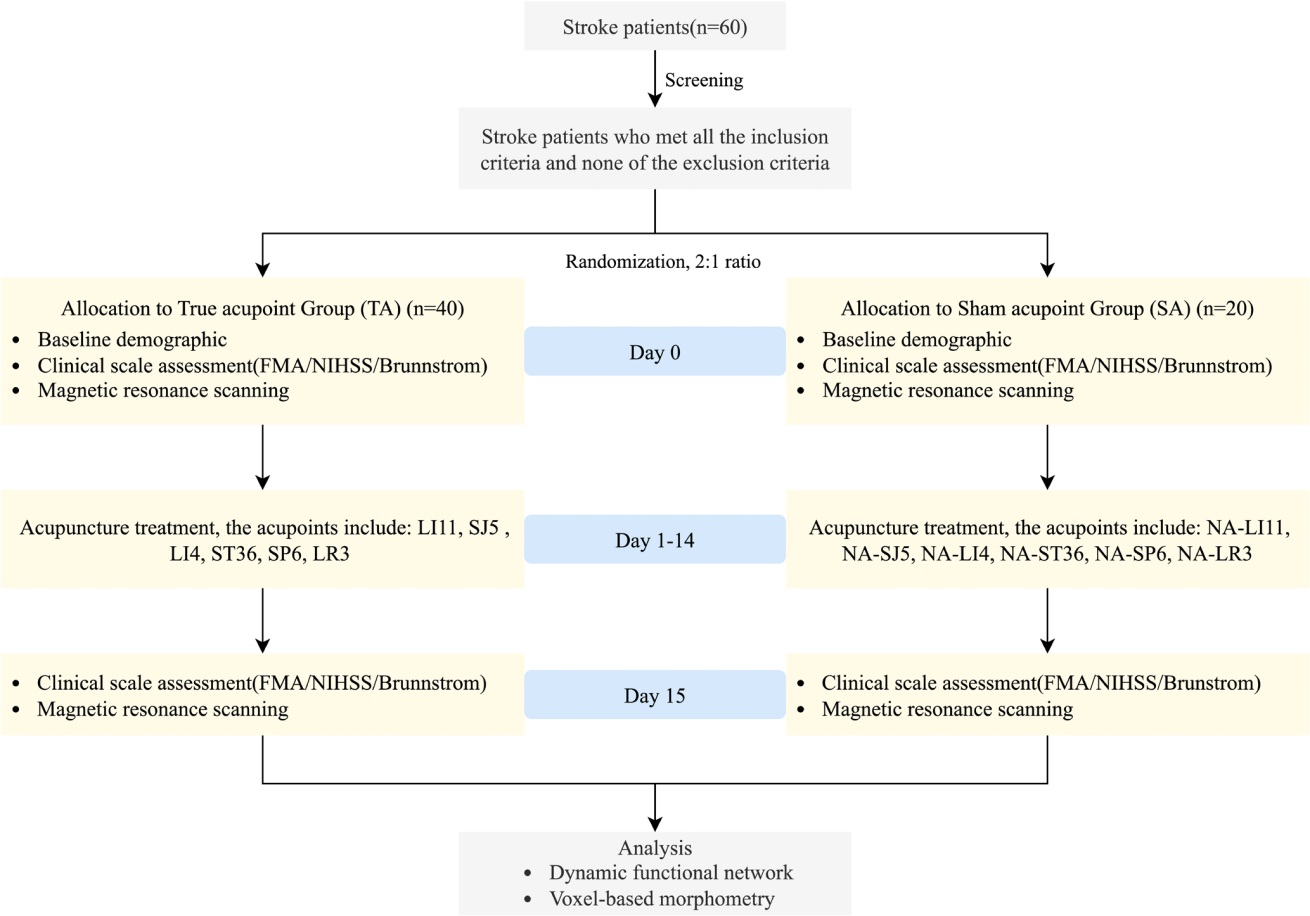
The Flowchart depicting the schedule of the trial. Abbreviation: FMA, Fugl‐Meyer Assessment; NIHSS, National Institutes of Health Stroke Scale.

This randomized, single‐blind, placebo‐controlled trial. Eligible patients with unilateral motor impairment within six weeks of an ischemic stroke were randomized to a true‐acupoint (TA) group receiving therapeutic acupuncture or a sham‐acupoint (SA) group receiving placebo acupuncture at non‐therapeutic locations. Randomization was carried out using a computer‐generated random number sequence. Allocation was concealed in sequentially numbered, sealed opaque envelopes. Upon enrollment, acupuncturists opened the envelopes to reveal the group assignment. Throughout the trial, only the acupuncturists were unblinded; patients, outcome assessors (including clinicians and MRI technicians), and data analysts remained blinded to the group allocation. The sensation data supporting blinding are presented in Table S2. All participants completed clinical assessments and multimodal MRI at baseline and after the intervention. The study design flowchart is presented in Figure 1.

### 2.2 Trial Participants

Eligible participants met all inclusion criteria: (1) diagnosis of ischemic stroke within the previous 6 weeks; (2) unilateral lesions confined to the centrum semiovale, corona radiata, or basal ganglia with predominant unilateral motor impairment; (3) right‐handedness prior to stroke; (4) age 35–80 years; (5) no psychotropic medication use in the preceding month.

Exclusion criteria comprised: (1) severe systemic disease (cardiac, renal, pulmonary, hepatic, or hematological); (2) active neuropsychiatric or infectious disorders; (3) MRI contraindications; (4) severe anatomical asymmetry or other significant pathologies on MRI; (5) prior participation in similar trials.

### 2.3 Sample Size

Previous neuroimaging studies have indicated that a sample size of approximately 16–32 participants is generally sufficient to achieve adequate statistical power when the cohort is highly homogeneous [14]. The present study enrolled patients presenting with unilateral motor impairment within six weeks of ischemic stroke, all of whom had lesions confined to the centrum semiovale, corona radiata, or basal ganglia—thereby ensuring a highly homogeneous study population. Accounting for anticipated dropout rates and exclusions due to image quality, we initially targeted a total of 60 participants in the experimental arm, with subsequent 2:1 randomization into the TA and SA groups [15].

### 2.4 Ethics Statement

This trial was conducted at Dongzhimen Hospital, Beijing University of Chinese Medicine, China, with ethics approval from the institutional review board (DMR100‐IRB‐068). All participants provided written informed consent.

### 2.5 Intervention

**FIGURE 2:**
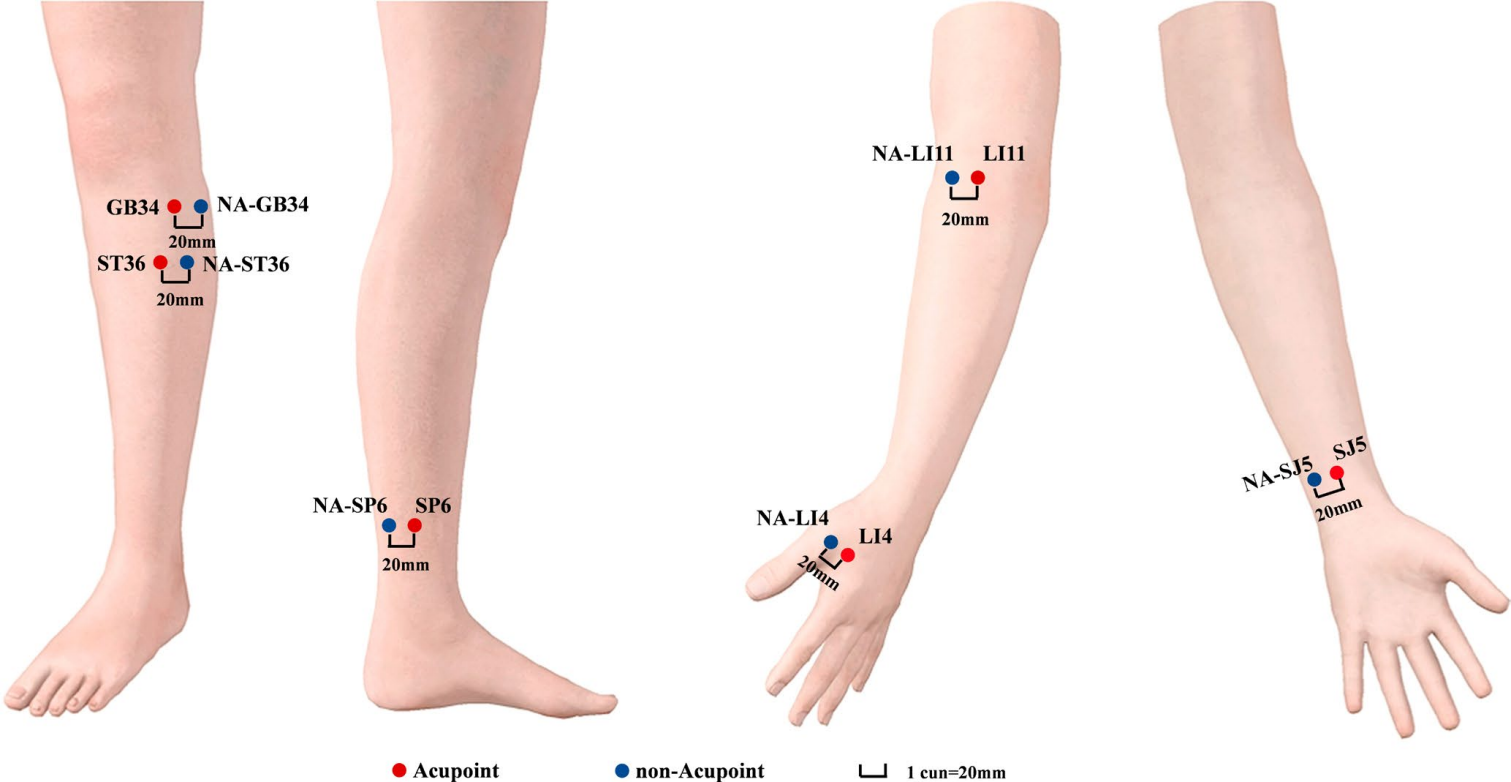
Locations of the acupoints and non‐acupoints are illustrated. The acupoint locations were measured using the cun unit (1 cun = 20 mm), which is defined as the width of the interphalangeal joint of the patient's thumb.

Standard pharmacotherapy for cerebrovascular disease was maintained in both groups. In the TA group, acupuncture was applied at clinically verified motor‐rehabilitation acupoints. The stimulated acupoints included the Quchi (LI11), Neiguan (SJ5), Hegu (SJ5), Zusanli (ST36), Sanyinjiao (SP6), and Yanglingquan (GB34). In contrast, the SA group underwent placebo acupuncture at non‐therapeutic locations, precisely 1 cun lateral to the corresponding true acupoints [16] (Figure 2). The details of the acupoint locations can be found in Table S1.

All participants received ten 30‐min sessions over two weeks (five sessions weekly). All treatments were administered by a licensed acupuncturist using sterile, disposable filiform needles (0.25 mm × 40 mm). For the TA group, needles were inserted perpendicularly (at a $90^{\circ }$) to a depth of 25 mm, following routine skin disinfection. The even reinforcing‐reducing manipulation (involving balanced twirling, rotating, lifting, and thrusting) was then applied at each acupoint for one minute to elicit the characteristic deqi sensation (soreness, numbness, distention, heaviness, or aching). The SA group received the same stimulation duration at non‐acupoints but without eliciting the deqi response.

### 2.6 MRI Data Acquisition

Functional images were acquired using a T2‐weighted gradient‐echo EPI sequence with the following parameters: TE = 30 ms, TR = 2,000 ms, flip angle = 90°, field of view = 225 × 225 mm^2^, matrix = 64 × 64, slice thickness = 3.5 mm.

High‐resolution anatomical images were obtained with a T1‐weighted sequence: TE = 2.53 ms, TR = 1,900 ms, flip angle = 9°, field of view = 250 × 250 mm^2^, matrix = 256 × 256, slice thickness = 1 mm.

During scanning, participants were maintained in audio‐visual isolation with head motion minimized using foam padding. The scanning order was as follows: Resting‐state fMRI (8 min 10 s) followed by T1‐weighted structural imaging (4 min 10 s).

### 2.7 Outcome Measurements

All subjects were assessed by the same neurologist on the day of enrollment and the day after completing acupuncture treatment, using the following scales: The Fugl‐Meyer Assessment (FMA) for motor impairment and recovery, the National Institutes of Health Stroke Scale (NIHSS) for neurological deficit (with lower scores indicating less impairment), and the Brunnstrom Scale for limb motor control.

### 2.8 Quality Control

Prior to MRI scanning, all participants were instructed to remain motionless, keep their eyes closed, and maintain a resting mental state without engaging in specific cognitive tasks. A case report form (CRF) was used to document each subject's baseline characteristics and motor assessment scores obtained after scanning. An independent researcher then verified the recorded data to ensure accuracy.

### 2.9 Processing Procedures for MRI Data

#### 2.9.1 Functional Image Data Preprocessing

Functional imaging data were preprocessed using the DPABI toolbox in SPM12 (http://www.fil.ion.ucl.ac.uk/spm) within MATLAB (MathWorks, Natick, Massachusetts, USA). The following preprocessing steps were performed on the fMRI data. The first ten volumes of each run were discarded to allow for scanner stabilization. Following slice‐timing correction, images were realigned to correct for head motion; participants exhibiting excessive displacement (> 3.0 mm or > 3.0°) were excluded. Functional images were then normalized to Montreal Neurological Institute (MNI) space using transformation parameters derived from each participant's T1‐weighted structural image, and resampled to 3 × 3 × 3 mm^3^ voxels. Spatial smoothing was applied with a 6‐mm full‐width at half‐maximum (FWHM) Gaussian kernel. Nuisance regressors, including the Friston‐24 motion parameters, global mean, and white matter signal, were regressed out. Finally, a temporal band‐pass filter (0.01–0.1 Hz) was applied to attenuate physiological noise.

#### 2.9.2 Construction of Multilayer Community Structure Network

Dynamic functional networks were constructed using the Dynamic BC toolbox in MATLAB [17]. These networks were based on the Anatomical Automatic Labeling (AAL) atlas [18], which divides the cerebral cortex into five resting‐state functional networks (RSNs): SMN, DMN, Attention Network (AN), Visual Network (VN), and Subcortical Network (SN).

**FIGURE 3:**
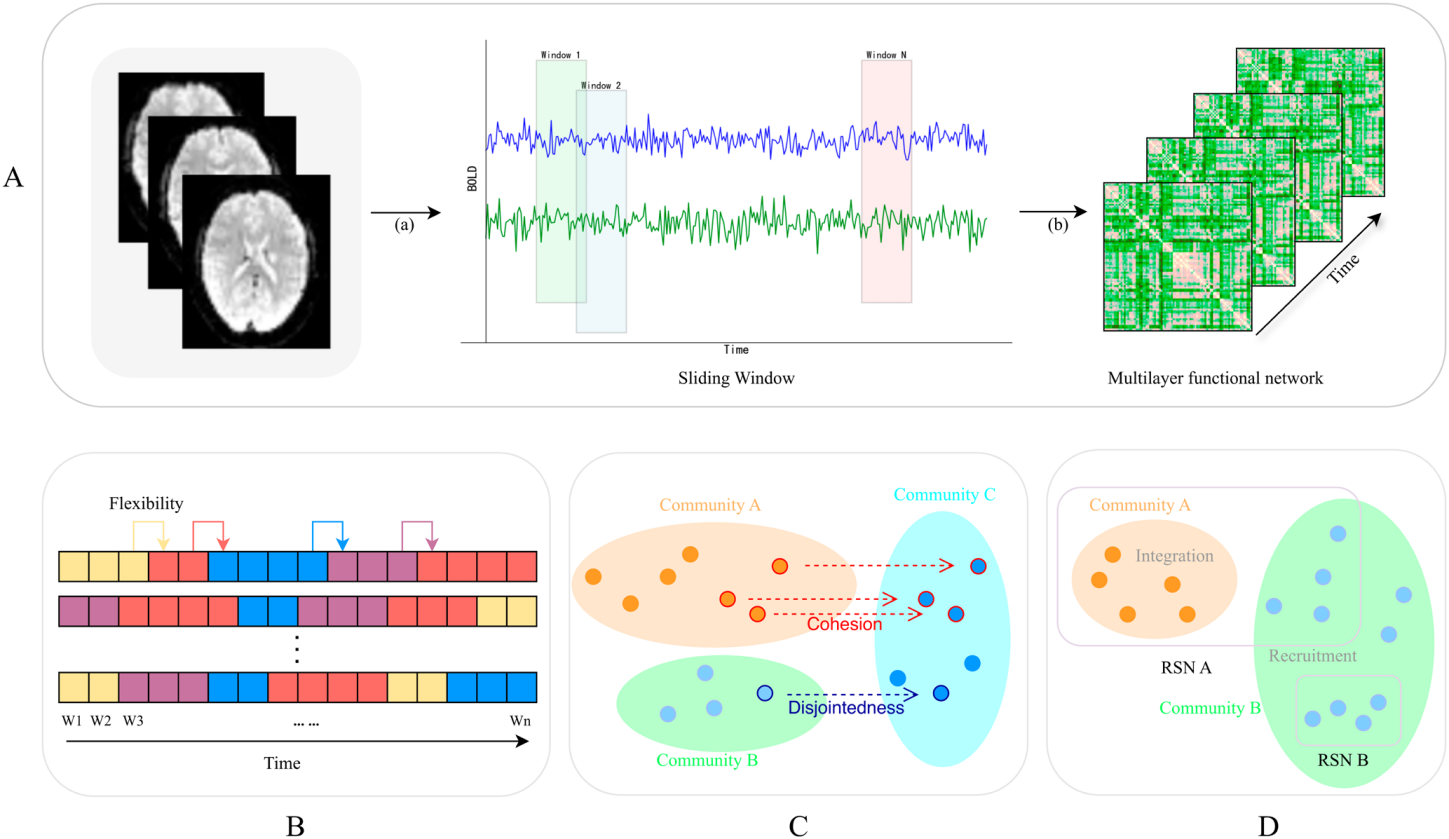
Data analysis flowchart. (A) Construction of Dynamic Multilayer Networks; (B) Calculation of flexibility; (C) Calculation of Cohesion and Disjointedness; (D) Calculation of Recruitment and Integration. Abbreviations: BOLD, Blood Oxygen Level‐dependent; RSN, Resting State Networks.

Following brain parcellation and regional time series extraction, dynamic functional connectivity was estimated using a sliding window approach (length = 50 TRs, step = 1 TR) [19, 20]. For each subject, a multilayer functional connectivity matrix of dimensions 90 × 90 × 120 (regions × regions × windows) was constructed by computing Pearson correlation coefficients. For multilayer community detection, we employed the GenLouvain toolbox with the default gamma (structural resolution parameter) and omega (inter‐layer coupling parameter) both set to 1 (https://github.com/GenLouvain/GenLouvain). This algorithm partitions nodes into distinct communities (modules) by optimizing a modularity function that favors strong intra‐community and weak inter‐community connections [21]. We then used flexibility to measure the frequency of module switching [22], cohesion, and disjointedness to measure the pattern of module switching [23]. Moreover, we assessed community transition probabilities via the recruitment and integration coefficient [24]. The procedures are detailed in the Table S1. The fMRI analysis process is shown in Figure 3.

#### 2.9.3 Structural Image Data Analysis

T1‐weighted structural images were first converted and aligned to the standard origin in the DPABI toolbox, followed by processing in CAT12 to calculate gray matter volume (GMV). CAT12 is a software package designed for brain structural analysis, which provides algorithms for optimizing gray matter segmentation [25]. Notably, it employs a projection‐based method to construct cortical maps that effectively address challenges such as partial volume effects and sulcal ambiguity, without requiring explicit sulcus reconstruction [25]. Preprocessing was conducted using the default pipeline in CAT12, which included the following steps: (1) bias field correction for intensity inhomogeneity; (2) tissue segmentation into gray matter, white matter, and cerebrospinal fluid; and (3) spatial normalization via the DARTEL (Diffeomorphic Anatomical Registration Through Exponentiated Lie Algebra) algorithm for spatial normalization to align individual images to the MNI coordinate space [26]. Following modulation, the gray matter images were segmented into 122 nodes defined by the AAL atlas. The mean GMV was calculated for each node within the TA and SA groups. Prior to statistical analysis, spatial smoothing was applied to the images of all subjects employing a Gaussian kernel of 8 mm FWHM.

### 2.10 Statistical Analysis

The normality of the difference scores (post‐treatment minus pre‐treatment) was assessed prior to parametric analyses. Within‐group comparisons of clinical scores and dynamic network metrics before vs. after treatment were conducted using paired‐samples *t*‐tests in Python3 for variables that met the normality assumption; variables that violated normality were analyzed using the Wilcoxon signed‐rank test. Statistical significance was set at *p* < 0.05. For dynamic network indices, the resulting *p*‐values were further corrected for multiple comparisons using the false discovery rate (FDR), and a corrected *P*_FDR_ < 0.05 was considered statistically significant. For the VBM analysis, a paired *t*‐test model in SPM12 was employed with a voxel‐level threshold of *p* < 0.001 and a cluster extent of 50 contiguous voxels. Significant clusters were then corrected using family‐wise error (FWE), with *P*_FWE_ < 0.05 considered significant.

## 3 Results

### 3.1 Participants

A total of 56 patients with ischemic stroke were randomized (2:1) to the TA (*n* = 38) or SA (*n* = 18) group. There were 6 study discontinuations (TA: *n* = 5; SA: *n* = 1) due to failure to complete the full acupuncture intervention or MRI scans. Following imaging data preprocessing, 4 patients were excluded due to excessive head movement during scanning (TA: *n* = 3; SA: *n* = 1). Ultimately, 46 patients were included in the MRI data analysis, with 30 in the TA group and 16 in the SA group. As summarized in Table 1, the two groups showed no significant differences in baseline demographic or clinical characteristics, including age, sex, disease duration, handedness, and initial clinical scores.

**TABLE 1:** Demographics, clinical variables of study participants.

Demographics	TA group (*n* = 30)	SA group (*n* = 16)	*p*	*t*‐value
Age (SD)	59.40 ± 11.54	61.13 ± 7.78	0.602	−0.525
Men/women	21/9	13/3	0.635	—
Course of disease (SD)	15.30 ± 11.78	15.56 ± 12.84	0.334	−0.336
Dominant hand (right)	30	16	—	—
FMA (SD)	67.10 ± 26.59	68.25 ± 32.46	0.900	−0.126
FMA‐Lower (SD)	26.60 ± 7.92	26.88 ± 7.94	0.913	−0.110
FMA‐Upper (SD)	40.47 ± 23.73	41.44 ± 25.23	0.900	−0.126
NIHSS(SD)	3.43 ± 2.55	4.50 ± 3.45	0.253	−1.158
Brunnstrom‐Upper (SD)	4.13 ± 2.05	4.13 ± 2.05	1.000	0.000
Brunnstrom‐Lower (SD)	4.57 ± 1.36	4.56 ± 1.62	0.993	0.009

Abbreviations: FMA, Fugl‐Meyer Assessment; NIHSS, National Institutes of Health Stroke Scale; SA, sham‐acupoint; SD, standard deviation; TA, true‐acupoint.

### 3.2 Comparison of Clinical Scales Before and After Acupuncture

Post‐treatment analysis revealed that the TA group exhibited significant increases in FMA, FMA‐Upper, FMA‐Lower, and Brunnstrom scores for both upper and lower limbs (all *p* < 0.001). The SA group also showed significant improvement in FMA (*p* = 0.015), FMA‐Upper (*p* = 0.035), and FMA‐Lower (*p* = 0.012) scores, whereas Brunnstrom upper limb (*p* = 0.333) and lower limb (*p* = 0.237) scores did not show statistical differences. Motor function improved significantly in both groups following acupuncture. However, the improvement in limb motor function was superior in the TA group compared to the SA group, as detailed in Table 2.

**TABLE 2:** Treatment outcomes of participants.

Clinical scales (SD)	TA group (*n* = 30)		*p*	*t*‐value	SA group (*n* = 16)		*p*	*t*‐value
	Before	After			Before	After		
FMA	67.10 ± 26.59	76.63 ± 24.41	**< 0.001**	−6.230	68.25 ± 32.46	59.40 ± 11.54	**0.015**	−2.760
FMA‐Lower	27.60 ± 7.92	30.17 ± 5.23	**< 0.001**	−3.864	26.88 ± 7.94	28.88 ± 6.34	**0.012**	−2.877
FMA‐Upper	40.47 ± 23.73	46.73 ± 21.09	**< 0.001**	−4.716	41.44 ± 25.23	46.38 ± 22.65	**0.035**	−2.318
NIHSS	3.43 ± 2.58	2.30 ± 2.42	**< 0.001**	4.852	4.50 ± 3.45	2.63 ± 2.74	**< 0.001**	4.496
Brunnstrom‐Upper	4.13 ± 2.05	4.80 ± 1.66	**< 0.001**	−3.959	4.13 ± 2.00	4.98 ± 1.83	0.333	−1.000
Brunnstrom‐Lower	4.57 ± 1.36	5.30 ± 1.04	**< 0.001**	−3.717	4.56 ± 1.62	4.88 ± 1.49	0.237	−1.232

*Note:* Bold values in Table 2 indicate statistical significance (*P* < 0.05).

Abbreviations: FMA, Fugl‐Meyer Assessment; NIHSS, National Institutes of Health Stroke Scale; SA, sham‐acupoint; SD, standard deviation; TA, true‐acupoint.

### 3.3 Effect of Acupuncture on Dynamic Functional Brain Network Indicators

**FIGURE 4:**
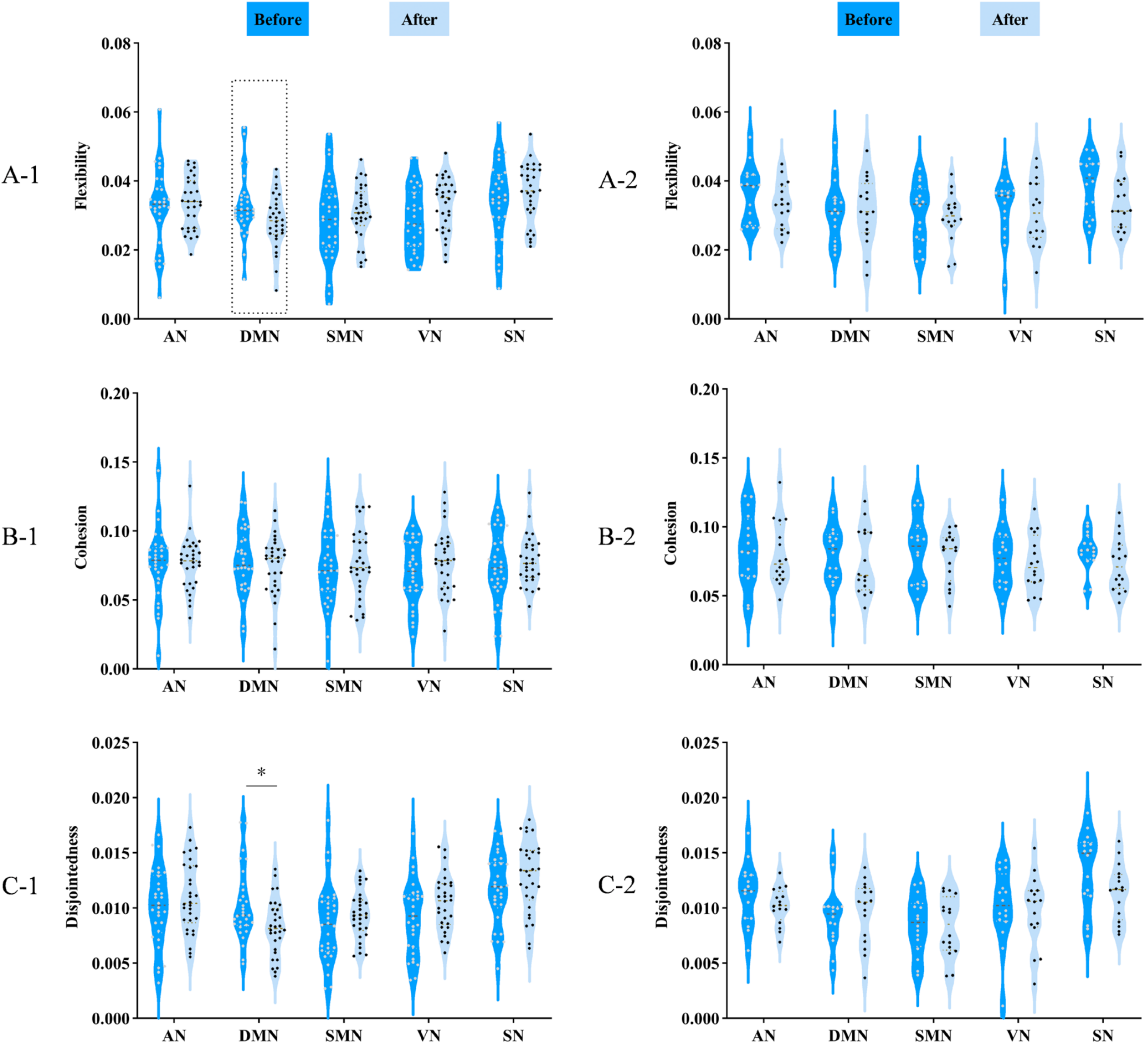
Changes in network transfer frequency following acupuncture treatment. (A) Changes in the flexibility of the two groups of patients before and after acupuncture treatment. (B) Changes in cohesion of the two groups of patients before and after acupuncture; (C) Changes in disjointedness of the two groups of patients before and after acupuncture. An asterisk (*) denotes that *P*_FDR_ < 0.05. Abbreviations: AN, Attention Network; DMN, Default Mode Network; SMN, Sensorimotor Network; VN, Visual Network; SN, Subcortical Network.

We used flexibility to quantify module switching frequency. In the TA group, the DMN exhibited a trend toward reduced flexibility post‐treatment (*P*_FDR_ = 0.094, *p* = 0.037, uncorrected). Further analysis of switching patterns revealed a reduction in DMN disjointedness in the TA group after treatment (*P*_FDR_ = 0.041). No significant changes in flexibility, disjointedness, or cohesion were observed in other RSNs of the TA group or in any RSNs of the SA group (all *P*_FDR_ > 0.05) (Figure 4).

**FIGURE 5:**
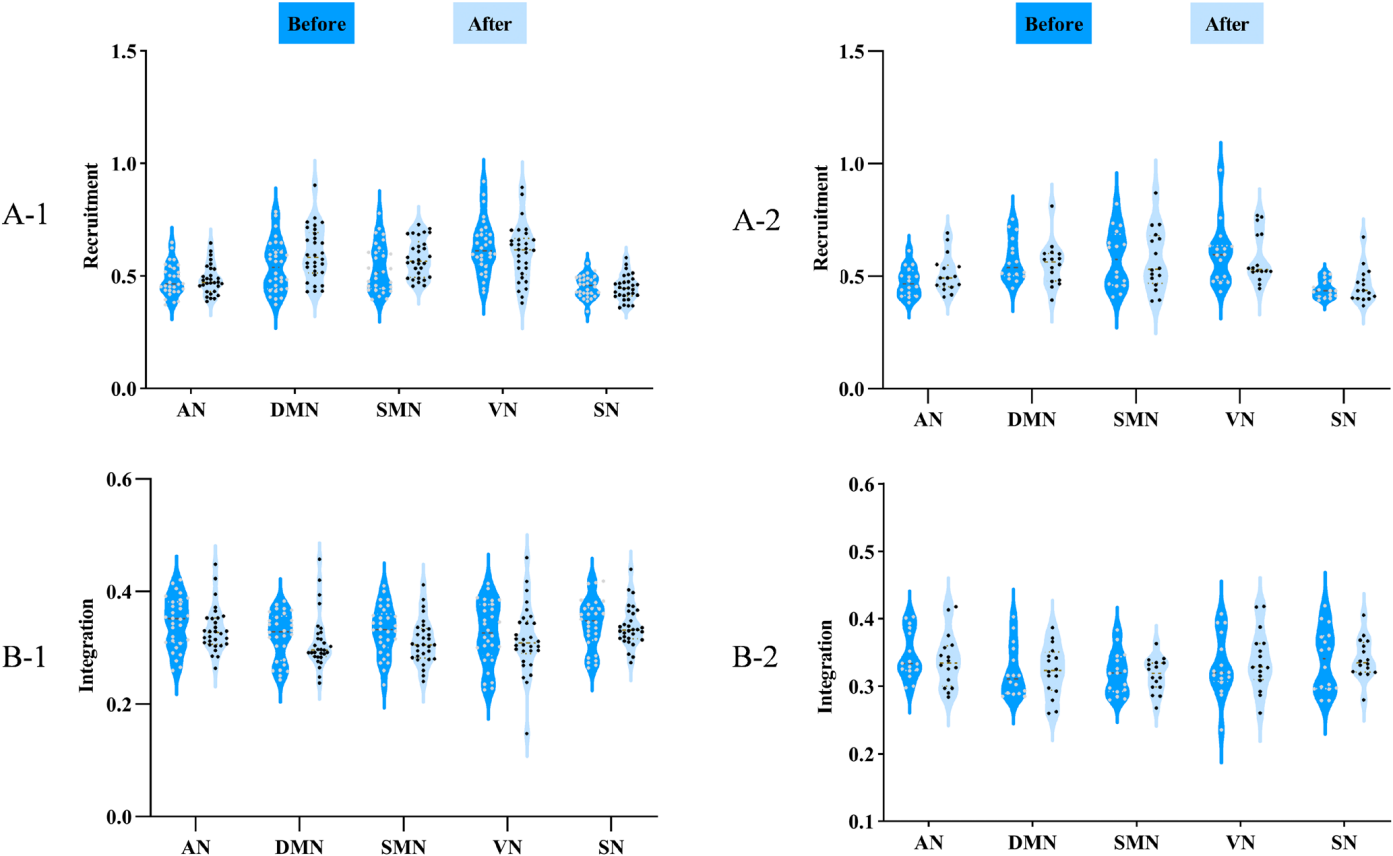
Changes in network transition probability following acupuncture treatment. (A) Changes in recruitment of the two groups of patients before and after acupuncture treatment. (B) Changes in the integration of the two groups of patients before and after acupuncture. Abbreviations: AN, Attention Network; DMN, Default Mode Network; SMN, Sensorimotor Network; VN, Visual Network; SN, Subcortical Network.

Furthermore, after acupuncture treatment, there was no statistically significant difference in the recruitment coefficient and integration coefficient in either the TA group or the SA group (all *P*_FDR_ > 0.05), as shown in Figure 5.

### 3.4 Effects of Acupuncture on Structural Morphology

In the TA group, the GMV of the right middle frontal gyrus, right postcentral gyrus, right angular gyrus, left superior parietal gyrus, left cerebellar Crus 1–2, 4–5, 7, bilateral middle occipital gyrus, superior temporal gyrus, inferior parietal angular gyrus, dorsal‐lateral superior frontal gyrus, and inferior frontal gyrus of the insula, as well as cerebellar region 10, increased significantly after acupuncture (mass level > 50, *P*_FWE_ < 0.05). No brain regions exhibited a significant decrease in GMV following treatment in either group (*P*_FWE_ > 0.05). In the SA group, no significant changes in GMV were observed after acupuncture treatment (*P*_FWE_ > 0.05). Detailed results are presented in Table 3. The visualization of GMV changes is shown in Figure S1.

**TABLE 3:** Localization of significant GMV increases following acupuncture in the TA group.

Cluster	Brain area	Peak MNI coordinates			Voxel	Peak *t*‐value	*p*
		X	Y	Z			
1	Right dorsolateral superior frontal gyrus	24	45	44	188,268	11.06	< 0.001
	Right middle frontal gyrus	39	60	0	—	—	—
2	Right postcentral gyrus	44	−30	63	7,461	10.66	< 0.001
	Right superior temporal gyrus	62	−44	23	—	—	—
	Angular gyrus of the right inferior parietal margin	51	−50	47	—	—	—
3	Right middle occipital gyrus	36	−69	24	791	9.94	< 0.001
	Left middle occipital gyrus	35	−69	24	—	—	—
4	Left superior parietal gyrus	−30	−51	66	3,036	9.23	< 0.001
5	Crus 1–2 areas of the left cerebellum	−6	−87	−23	186	8.05	< 0.001
6	Angular gyrus of the left parietal inferior margin	−57	−35	47	1,568	7.51	0.001
7	Inferior frontal gyrus in the left operculum	−23	24	−14	64	7.39	0.005
8	Left dorsolateral superior frontal gyrus	−18	29	51	302	7.19	< 0.001
9	Inferior frontal gyrus in the right operculum	56	30	−8	176	6.68	< 0.001
10	Area 7 of the left cerebellum	−42	−41	−39	262	6.81	< 0.001
	Area 10 of the left cerebellum	−26	−38	−42	—	—	—
11	Area 10 of the right cerebellum	29	−41	−42	115	6.80	0.002
12	Left superior temporal gyrus	−63	−45	23	127	6.65	0.001
13	Right corner back	36	−65	53	52	6.19	0.006
14	Areas 4–5 of the left cerebellum	−9	−44	−12	55	6.14	0.006

Abbreviations: GMV, gray matter volume; MNI, Montreal Neurological Institute.

### 3.5 Correlation Analysis

**FIGURE 6:**
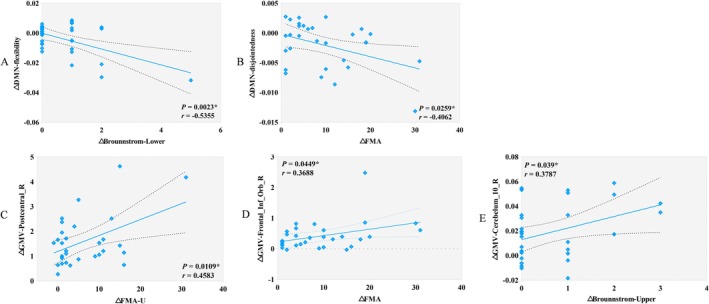
Correlation analysis results. (A) The reduction in DMN flexibility after TA treatment was negatively correlated with improvement in Brunnstrom lower‐limb scores. (B) The reduction in DMN disjointedness was negatively correlated with improvement in FMA scores. (C) The increase in GMV in the right postcentral gyrus was positively correlated with improvement in FMA upper‐limb scores. (D) The increase in GMV in the right frontal operculum (inferior frontal gyrus) was positively correlated with improvement in FMA scores. (E) The increase in GMV in the right cerebellar area 10 was positively correlated with improvement in Brunnstrom upper‐limb scores. Abbreviations: DMN, Default Mode Network; GMV, Gray Matter Volume; FMA, Fugl‐Meyer Assessment; FMA‐U, FMA‐Upper.

Correlation analysis showed that changes in flexibility and disjointedness of the DMN after TA intervention were negatively correlated with improvements in clinical scale scores. Additionally, a positive correlation was found between the improvement of FMA score and GMV increases in the right operculum inferior frontal gyrus, right postcentral gyrus, and right cerebellar region 10 after TA intervention, as shown in Figure 6.

### 3.6 Adverse Events

No adverse events occurred in either group.

## 4 Discussion

Motor function impairment is one of the most common symptoms after ischemic stroke. Post‐stroke recovery is fundamentally supported by brain plasticity, which involves cortical remapping and the reorganization of redundant neural connections into new structural and functional circuits [27]. This neuroplastic capacity forms a crucial theoretical basis for rehabilitation treatment. However, the neural mechanisms underlying motor recovery are highly complex, encompassing functional and structural reorganization throughout the brain. sMRI provides high‐resolution anatomical visualization of neuroanatomical changes, whereas fMRI captures the dynamics of functional recovery via BOLD signals [28]. Integrating these complementary modalities provides a comprehensive framework for elucidating the central mechanisms of acupuncture in treating post‐stroke motor impairment from both anatomical and functional perspectives.

This multimodal neuroimaging study investigated the central mechanisms of acupuncture in treating post‐stroke hemiparesis. The main findings were as follows: First, acupuncture alleviated neurological deficits and promoted motor recovery. Second, neuroimaging revealed that acupuncture normalized hyperactivity and disjointedness of the DMN and restored regional cortical morphology in areas related to sensorimotor processing, cognition, and attention. These modulations may improve motor initiation, execution, control, and coordination, representing a potential central mechanism underlying acupuncture's therapeutic effect. Third, compared with SA, TA more effectively normalized aberrant RSNs and modulated specific gray matter structures, and these neural changes correlated with the recovery of limb motor function, potentially underpinning the superior therapeutic efficacy of TA.

### 4.1 Clinical Outcomes

Before treatment, clinical scale scores did not differ between the TA and SA groups, confirming comparable baseline characteristics. After acupuncture treatment, patients in the TA group showed significant increases in FMA, FMA‐Upper, FMA‐Lower, Brunnstrom‐Upper, and Brunnstrom‐Lower scores compared to baseline. Although the SA group also exhibited post‐treatment improvement in FMA scores, no significant change was observed in Brunnstrom scores for either upper or lower limbs, indicating that TA offers a distinct advantage in ameliorating limb spasticity. The modest improvement in the SA group may be explained by non‐specific placebo effects associated with needle insertion at a non‐therapeutic site, consistent with prior reports [29]. In addition, both groups received the concurrent standard pharmacotherapy during hospitalization; therefore, patients in the SA group also showed improvement in motor function.

### 4.2 Modulation of the Functional Brain Network by Acupuncture

Post‐stroke brain remodeling extends beyond the peri‐lesional region to drive functional reorganization throughout the brain [30], a process fundamentally driven by the reorganization of functional networks [31]. Analysis of the dynamic network allows quantification of how brain remodeling occurs across subnetworks after stroke. In the present study, we observed that TA reduced DMN disjointedness and showed a trend toward decreased flexibility. The disjointedness metric quantifies the temporal stability of network architecture: Lower values indicate that DMN nodes maintain more stable module allegiance over time, reflecting greater coherence and reduced fragmentation of the network. Notably, this finding contrasts with our previous observation that stroke patients exhibit significantly greater flexibility and disjointedness in the DMN than healthy subjects [32], suggesting that acupuncture may help normalize these aberrant network dynamics. The DMN supports core cognitive functions such as emotional regulation, self‐awareness, and attention maintenance [33]. From a neurophysiological perspective, excessive DMN fragmentation, characterized by high disjointedness, may reflect inefficient allocation of cognitive resources. Conversely, the restored cohesion observed following acupuncture may enable more efficient network interactions, potentially reducing cross‐network interference and freeing cognitive resources for goal‐directed behaviors. In patients with subcortical stroke, functional connectivity both within the DMN and between the DMN and other networks is significantly altered [34]. Such alterations are observed even in stroke patients without cognitive impairment [35]. Since the cognitive system contributes to motor execution by monitoring performance, planning movements, and integrating sensorimotor feedback [36], DMN dysfunction may impede motor recovery, particularly in tasks that demand high attentional load, such as coordinated grasping or obstacle avoidance. Given that sustained hyperactivity often signifies poor recovery, while normalization of activity predicts positive outcomes [37, 38], our findings indicate that acupuncture facilitates recovery by specifically modulating and normalizing the post‐stroke aberrant dynamic reorganization of the DMN.

Our correlation analyses further showed that reductions in flexibility and disjointedness in the DMN following TA intervention were negatively correlated with improvements in clinical motor scores. Our results demonstrate that acupuncture can normalize the aberrant dynamic reorganization of the DMN following stroke, which may be beneficial in processing motor tasks, particularly when the sensorimotor network was severely damaged. Recent studies have also confirmed the relationship between the DMN and motor function recovery after stroke [39]. A study exploring brain networks in hemiplegic patients after stroke, using Granger causality analysis, found that acupuncture could modulate the DMN, turning it into a relay station for information transmission [40]. Together, these findings suggest that acupuncture may facilitate motor recovery by correcting dysfunctional DMN patterns, thereby improving the cognitive preparation and execution of motor behavior.

### 4.3 Regulation of GMV by Acupuncture

The brain functions as a complex, interconnected network system. Consequently, focal subcortical damage can induce remote effects in cortical regions via their structural connections. Subcortical infarction is known to trigger widespread GMV reduction throughout the brain, with specific secondary degeneration occurring in regions directly or indirectly connected to the lesion. This remote GMV loss in patients with post‐stroke hemiplegia may arise from multiple mechanisms, including axonal degeneration, demyelination, reduced regional cerebral blood flow, and metabolic impairment [41, 42, 43]. In the present study, we observed GMV increases following acupuncture across a distributed set of brain regions involved in motor execution, including frontal, parietal, temporal, occipital, and cerebellar areas. It is worth noting that MRI‐derived GMV measures do not directly equate to confirmed microstructural or cellular plasticity. Rather, they serve as potential imaging markers of recovery‐related structural adaptation.

Several of the regions showing GMV increases have well‐established roles in motor control. Within the frontal lobe, the superior, middle, and inferior frontal gyri are critically involved in motor execution. The superior frontal gyrus, as part of the supplementary motor area, connects to the pyramidal tract and contributes to the regulation of bilateral movements. The middle frontal gyrus supports cognitive control [44] and early motor learning [45], especially during locomotion [46], visuomotor sequencing [47], and attentional tasks [48]. Furthermore, the prefrontal cortex gives rise to the frontopontine tract, forming part of the corticocerebellar circuit that modulates movement speed, amplitude, and coordination. Evidence indicates reduced GMV in frontal regions after stroke [49, 50]. Studies have reported GMV reductions in the bilateral frontal lobes of patients with upper limb motor dysfunction [51], which highlight the essential role of these areas in translating higher‐order commands into motor actions.

Parietal regions also exhibited structural changes. The superior parietal gyrus contributes to retrieving past situational information, supporting the formulation and execution of goal‐directed actions. The postcentral gyrus, which forms the primary somatosensory cortex, processes somatosensory input via thalamic projections. Rehabilitation interventions such as constraint‐induced movement therapy and virtual training have been shown to increase GMV in the affected sensorimotor cortex of stroke patients [52, 53]. Furthermore, the posterior parietal cortex is divided by the intraparietal sulcus into the superior parietal lobule (including the superior parietal gyrus) and the inferior parietal lobule (including the angular gyrus), regions that are critically involved in sensorimotor integration, spatial attention, and early motor planning [54]. Beyond parietal areas, temporal and occipital regions also showed GMV changes. The temporal lobe gives rise to efferent pathways such as the tectal and geniculocortical tracts, which support multisensory integration. The superior temporal gyrus, a core vestibular region, contributes to postural stability, and damage here can lead to balance deficits. The middle occipital gyrus, located in the medial parieto‐occipital cortex, is involved in spatial cognition and visuomotor processing [55]. Visual input is known to facilitate motor performance; for example, balance is better maintained with eyes open than closed. Voluntary movement planning and execution depend on visually guided spatial localization and subsequent cortical motor processing [56]. Notably, the superior parietal lobule, temporal auditory cortex, and occipital visual cortex are all components of a broader sensorimotor network that participates in voluntary movement regulation [57]. Cerebellar contributions are equally important. Extensive cerebro‐cerebellar connections via white matter tracts underlie the involvement of cortico‐cerebellar circuits in motor learning and sensorimotor integration [58]. The anterior cerebellar lobe (lobules I–V) and portions of the posterior lobe (lobules VI and VIII) are primarily sensorimotor in function and serve as key hubs for movement coordination [59]. Therefore, accurate motor execution relies not only on primary and non‐primary motor cortices, but also on visuospatial processing and motor planning. The observed GMV increases in the middle occipital gyrus, superior parietal gyrus, superior temporal gyrus, angular gyrus, and posterior cerebellum suggest that acupuncture promotes structural adaptations that compensate for stroke‐induced circuit disruption, potentially supporting the subsequent restoration of motor function. Consistent with our observations, previous fMRI studies have reported altered low‐frequency fluctuation (ALFF) in frontal, parietal, cerebellar, and other motor‐ and visual‐related regions following stroke [60, 61]. Changes in nodal degree centrality (DC) within the occipital cortex have also been linked to motor performance in patients with hemiplegia [62]. Acupuncture can promote functional reorganization and improve limb motor function by bidirectionally normalizing aberrant functional connectivity between the primary motor cortex and the cerebellum [63].

Taken together, our findings indicate that acupuncture‐induced motor recovery is associated with structural plasticity in the sensorimotor, visual, auditory, and cerebellar motor cortices. Moreover, we observed a positive correlation between the extent of motor improvement and increases in GMV in the right frontal operculum/inferior frontal gyrus, postcentral gyrus, and cerebellar region 10 following acupuncture. These results suggest that acupuncture may promote recovery from limb motor dysfunction by selectively enhancing structural plasticity within this distributed sensorimotor integration circuitry.

Compared with the SA, TA specifically normalized aberrant RSNs and modulated GMV across multiple brain regions in patients with hemiplegia. These neuroplastic changes were correlated with motor recovery, suggesting a potential mechanism underlying the therapeutic specificity of verum acupoint stimulation. Importantly, although both groups exhibited clinical improvement, the distinct neural reorganization observed only in the TA group confirms that acupuncture exerts measurable and disease‐relevant effects on the central nervous system beyond non‐specific or placebo responses.

## 5 Limitations

Several limitations of this study should be considered. First, ischemic stroke is a clinically heterogeneous disease. Due to the differences in lesion location, disease course, and severity of neurological deficit, considerable variability in brain reorganization patterns can occur. Therefore, we controlled for key variables including disease duration, lesion site, and core symptoms to enhance sample homogeneity. Second, although the current sample size is consistent with many neuroimaging studies, a larger cohort would enable more robust analyses and potentially allow for subgrouping based on clinical severity in future investigations. Third, we acknowledge the absence of a statistically significant group × time interaction. This finding should be interpreted with caution, as both groups received concurrent standard pharmacotherapy, which likely contributed to clinical improvements in the SA group. Nevertheless, the within‐group neuroplastic changes observed in the TA group and their correlation with motor recovery support our primary mechanistic hypothesis. Future confirmatory trials with larger sample sizes are warranted to formally test group × time interactions. Fourth, the absence of long‐term follow‐up data limits our ability to determine the durability of the observed neuroplastic changes and their sustained impact on motor recovery. Future studies with extended longitudinal assessments are warranted to establish whether acupuncture‐induced brain reorganization translates into lasting functional benefits.

## 6 Conclusion

In summary, by integrating static structural and dynamic functional metrics, this study demonstrates that acupuncture significantly improved clinical motor symptoms in patients with ischemic stroke. Functionally, it normalized aberrant activity and disjointedness of the DMN, and structurally, it counteracted stroke‐related GMV reduction. These specific neural changes were correlated with recovery of limb motor function, suggesting a central mechanism through which acupuncture promotes motor restoration after stroke.

## Author Contributions

**Xin Yu** (first author) performed the fMRI data analysis and statistical tests and drafted the manuscript. **Yihuai Zou** designed the study and supervised the research. **Nana Zhao** co‐supervised the research. **Yuchen Liu** conducted additional statistical analysis. **Shizhong Zheng**, **Dongtao Luo**, and **Qianji Chen** administered the acupuncture treatments. **Fufu Zeng**, **Yanfei Jia**, and **Jiao Chen** conducted the clinical assessments. All authors contributed to the interpretation of the results and approved the final version for publication.

## Funding

This work was supported by the Scientific Research Project of the Guangdong Provincial Administration of Traditional Chinese Medicine (20262046) and the National Natural Science Foundation of China (82174331).

## Conflicts of Interest

The authors declare no conflicts of interest.

## Supporting information


**Table S1:** Location of acupoints and non‐acupoints.
**Table S2:** Non‐Specific Sensations Associated with Needle Insertion.
**Figure S1:** Localization of GMV increases following acupuncture. Yellow regions signify areas of significant GMV increase post‐treatment. The color bars indicate the overlap rate across subjects. Abbreviations: GMV, Gray Matter Volume.

## Data Availability

The data that support the findings of this study are available from the corresponding author upon reasonable request.
