# Supplementary material for: Neuroplastic Mechanisms of Acupuncture in Post‐Stroke Motor Recovery: A Randomized Multimodal MRI Trial
Source: CNS Neurosci Ther. 2026 Jun 3;32(6):e70955. doi: 10.1002/cns.70955 (PMC13239543; doi:10.1002/cns.70955)
Supplement: Supplementary file 1 — Table S1: Location of acupoints and non‐acupoints. Table S2: Non‐Specific Sensations Associated with Needle Insertion. Figure S1: Localization of GMV increases following acupuncture. Yellow regions signify areas of significant GMV increase post‐treatment. The color bars indicate the overlap rate across subjects. Abbreviations: GMV, Gray Matter Volume. [file CNS-32-e70955-s001.docx]

**Methods**

- 1. **Location of acupoints and non-acupoints**

The specific locations of the acupoints and non-acupoints are shown in Supplementary Table 1.

**1.2 Construction of Multilayer Community Structure Network**

The corresponding multilayer modularity quality function is defined as:

$$\begin{aligned} Q=\frac{1}{2\mu}\sum_{ijlr} \left( \left( A_{ijl}-\gamma_{l}\cdot P_{ijl} \right)\delta_{lr}+\delta_{ij}\omega_{jlr} \right)\delta\left( g_{il},g_{jr} \right)\#\left( 1 \right) \end{aligned}$$

The modularity metric $Q$ reflects partition quality, where higher values indicate more meaningful community segregation. Within the modularity function $Q$, $\mu$ represents the global edge weight summation across the multilayer network. $A_{ijl}$ quantifies the connection strength between nodes $i$ and $j$ in layer $l$. The Newman-Girvan null model provides the expected weight matrix $P_{ijl}$. $g_{il}$ and $g_{jr}$ denote module membership for node $i$ in layer $l$ and node $j$ in layer $r$, respectively. We fix the structural resolution parameter $\gamma_{l}$ = 1 and temporal coupling parameter $\omega_{jlr}$ = 1 for all layers to prevent algorithmic bias toward predetermined modular scales or temporal dynamics^[1]^. Given the algorithm's stochastic nature and propensity for local optima, we conduct 50 independent optimizations per temporal network^[2]^, retaining the partition with maximal $Q$ for subsequent analysis.

**1.3 Multilayer Temporal Dynamics Analysis**

Multilayer modularity analysis assigns brain regions to modules within successive temporal windows. To quantify the dynamic reconfiguration of these modules, we defined node flexibility $f_{i}$ as the normalized frequency of module assignment changes for node $i$ between consecutive time windows, following established methods^[1]^. This is formalized as:

$$\begin{aligned} f_{i}=1-\frac{1}{T-1}\sum_{s=1}^{T-1} \delta\left( g_{i,s},g_{i,s+1} \right)\#\left( 2 \right) \end{aligned}$$

Here, $T$ represents the time window. If node $i$ remains in the same module in both layer $s$ and layer $s+1$, then $\delta\left( g_{i,s},g_{i,s+1} \right) = 0$, else,$\delta\left( g_{i,s},g_{i,s+1} \right) = 1$.

The variability in flexibility was further characterized using two distinct metrics: cohesion and disjointedness^[3]^. Cohesion quantifies the propensity of a node to change its community assignment synchronously with others. It can be described as:

$$\begin{aligned} \Omega_{i}=\sum_{j\neq i} M_{ij}\#\left( 3 \right) \end{aligned}$$

The cohesion matrix, denoted as $M$, $M_{ij}$records the frequency with which nodes $i$ and $j$ concurrently reconfigure to the same functional community.

In contrast, disjointedness measures a node switching its community independently without any other nodes, which can be described as:

$$\begin{aligned} \Delta_{i}=\frac{g_{i}^{isk}}{L-1}\#\left( 4 \right) \end{aligned}$$

$g_{i}^{isk}$ represents that node $i$ is the only node transitioning from community $s$ to community $k$,$\Delta_{i}$quantifies the probability of node $i$ executing such an isolated transition across all $L-1$ potential community changes.

We assessed community transition probabilities via the recruitment and integration coefficient^[4]^. The recruitment coefficient quantifies the tendency for nodes within the same RSN to co-assign to identical modules, reflecting intra-network alignment. Conversely, the integration coefficient measures a node's propensity to share modular membership with nodes from distinct RSNs, indicating inter-network integration.

The recruitment coefficient for node $i$ within functional network $s$ is calculated as:

$$\begin{aligned} R_{i}^{s}=\frac{1}{n_{s}}\sum_{j\in s} P_{ij}\#\left( 5 \right) \end{aligned}$$

The integration coefficient for node $i$ within functional network $s$ is calculated as:

$$\begin{aligned} I_{i}^{s}=\frac{1}{N-n_{s}}\sum_{j\notin s} P_{ij}\#\left( 6 \right) \end{aligned}$$

In the two equations, $n_{s}$ denotes the number of nodes within network $s$. Critically, dynamic recruitment and integration metrics are distinct from traditional functional or structural connectivity strength in a mathematical sense. Instead, they quantify the time-varying probability of a brain region's modular co-assignment with its native network (recruitment) or foreign networks (integration).

**Results**

**1.1 Changes in Gray Matter Volume**

GMV increases following acupuncture were visualized using XjView software in MATLAB (Supplementary Fig. 1).

**1.2 Non-specific Acupuncture Sensation Survey**

No significant differences were observed between the TA and SA groups in any type of non-specific needling sensation (all *P* > 0.05). Both groups experienced comparable rates of somatosensory stimuli associated with needle insertion, supporting the maintenance of participant blinding (Supplementary Table 2).

**References:**

[1] HARLALKA V, BAPI R S, VINOD P K, et al. Atypical Flexibility in Dynamic Functional Connectivity Quantifies the Severity in Autism Spectrum Disorder [J]. Front Hum Neurosci, 2019, 13: 6.

[2] BASSETT D S, PORTER M A, WYMBS N F, et al. Robust detection of dynamic community structure in networks [J]. Chaos, 2013, 23(1): 013142.

[3] GARCIA J O, ASHOURVAN A, MULDOON S F, et al. Applications of community detection techniques to brain graphs: Algorithmic considerations and implications for neural function [J]. Proc IEEE Inst Electr Electron Eng, 2018, 106(5): 846-67.

[4] WU K, JELFS B, NEVILLE K, et al. Dynamic Reconfiguration of Brain Functional Network in Stroke [J]. IEEE J Biomed Health Inform, 2024, 28(6): 3649-59.

Supplementary Table 1: Location of acupoints and non-acupoints

| **Acupoints** | **Acupoints Location** | **No-acupoints Location** |
| --- | --- | --- |
| LI11 | Lateral end of the cubital crease, depression anterior to the lateral epicondyle of the humerus | At the lateral terminus of the elbow crease. Midpoint of the line connecting the humeral lateral epicondyle and the crease, 1 cun radial offset |
| SJ5 | 2 cun proximal to the dorsal wrist crease. In the intertendinous depression between the extensor carpi ulnaris and the extensor digitorum | Dorsal forearm, 2 cun proximal to the dorsal wrist crease. In the depression between the ulna and radius, offset 1 cun radially |
| LI4 | On the dorsum of the hand. Midpoint of the first intermetacarpal space, over the maximal prominence of the first dorsal interosseous muscle | Dorsum of the hand. Midpoint between first and second metacarpals, adjacent to ulnar side of first metacarpal, 1 cun from LI4 |
| ST36 | Anterolateral leg. 3 cun below the inferior patellar border. One finger-breadth lateral to the anterior tibial crest, in depression between tibialis anterior and extensor digitorum longus | Anterolateral lower leg. 3 cun distal to the lateral knee depression. Lateral to the anterior tibial crest, 1 cun lateral to ST36 |
| SP6 | Medial leg, 3 cun proximal to the medial malleolar apex. In the depression posterior to the medial tibial border | Medial calf. 3 cun proximal to the medial malleolar tip. 1 cun posterior to medial tibial border |
| GB34 | Lateral leg. In the depression anterior and inferior to the fibular head | Lateral leg, in the depression anterior and inferior to the fibular head. Offset 1 cun laterally |

*The acupoint locations were measured using the cun unit (1 cun = 20 mm), which is defined as the width of the interphalangeal joint of patient’s thumb.

Supplementary Table 2: Non-Specific Sensations Associated with Needle Insertion

| **Sensation Type** | **TA Group (n=30)** | **SA Group (n=16)** | ***P* Value** |
| --- | --- | --- | --- |
| Sharp/stabbing pain | 12 (40.0%) | 6 (37.5%) | 0.869 |
| Dull ache | 10 (33.3%) | 5 (31.3%) | 0.888 |
| Tingling sensation | 9 (30.0%) | 5 (31.3%) | 0.930 |
| Pressure sensation | 7 (23.3%) | 4 (25.0%) | 0.899 |
| No sensation | 2 (6.7%) | 2 (12.5%) | 0.601^i^ |

*Data are presented as number of participants reporting each sensation (percentage). Participants could report multiple sensations*.* ⁱFisher's exact test was used due to expected cell count < 5.


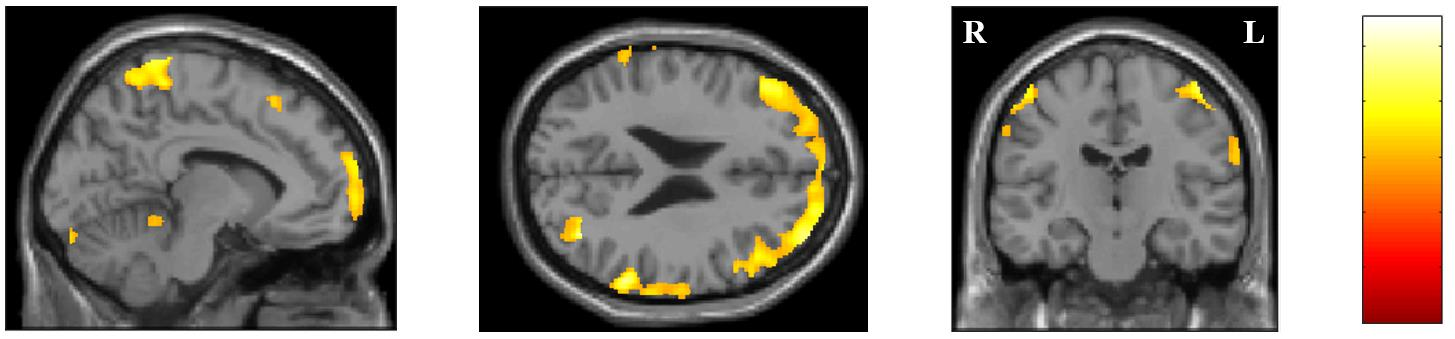


**Supplementary Fig. 1** Localization of GMV increases following acupuncture. Yellow regions signify areas of significant GMV increase post-treatment. The color bars indicate the overlap rate across subjects. Abbreviations: GMV, Grey Matter Volume.
